# Global and population-specific association of MTHFR polymorphisms with preterm birth risk: a consolidated analysis of 44 studies

**DOI:** 10.1186/s12884-025-07378-6

**Published:** 2025-03-01

**Authors:** Maryam Vafapour, Hanieh Talebi, Mahsa Danaei, Maryam Yeganegi, Sepideh Azizi, Seyed Alireza Dastgheib, Reza Bahrami, Melina Pourkazemi, Fatemeh Jayervand, Amirhossein Shahbazi, Heewa Rashnavadi, Ali Masoudi, Amirmasoud Shiri, Hossein Neamatzadeh

**Affiliations:** 1 Department of Pediatrics, Firoozabadi Clinical Research Development Unit, Iran University of Medical Sciences, Tehran, Iran; 2 Clinical Research Development Unit, Fatemieh Hospital, Hamadan University of Medical Sciences, Hamadan, Iran; 3 Department of Obstetrics and Gynecology, School of Medicine, Iran University of Medical Sciences, Tehran, Iran; 4 Department of Obstetrics and Gynecology, School of Medicine, Iranshahr University of Medical Sciences, Iranshahr, Iran; 5 Shahid Akbarabadi Clinical Research Development Unit, Iran University of Medical Sciences, Tehran, Iran; 6 Department of Medical Genetics, School of Medicine, Shiraz University of Medical Sciences, Shiraz, Iran; 7 Neonatal Research Center, Shiraz University of Medical Sciences, Shiraz, Iran; 8 Student Research Committee, School of Medicine, Iran University of Medical Sciences, Tehran, Iran; 9 Student Research Committee, School of Medicine, Ilam University of Medical Sciences, Ilam, Iran; 10 Student Research Committee, School of Medicine, Tehran University of Medical Sciences, Tehran, Iran; 11 Mother and Newborn Health Research Center, Shahid Sadoughi University of Medical Sciences, Yazd, Iran

**Keywords:** Preterm birth, MTHFR gene, Genetic polymorphisms, Ethnic variability, Pregnancy complications

## Abstract

**Background:**

This study investigates the relationship between polymorphisms in the MTHFR gene and the risk of preterm birth (PTB).

**Methods:**

A comprehensive literature review was conducted using databases such as PubMed, Web of Science, and CNKI, with the search finalized on January 1, 2025. The review specifically targeted studies published prior to this date, utilizing relevant keywords and MeSH terms associated with PTB and genetic factors. Inclusion criteria encompassed original case-control, longitudinal, or cohort studies, with no limitations on language or publication date. Associations were quantified using odds ratios (ORs) and 95% confidence intervals (CIs) via Comprehensive Meta-Analysis software.

**Results:**

The analysis included 44 case-control studies comprising 7,384 cases and 51,449 controls, extracted from 28 publications in both English and Chinese. Among these studies, 29 focused on the MTHFR C677T polymorphism, while 15 examined the MTHFR A1298C variant. Pooled results demonstrated a significant association between the MTHFR C677T polymorphism and PTB under five genetic models: allele (C vs. T; OR = 1.303, 95% CI 1.151–1.475, *p* ≤ 0.001), homozygote (CC vs. AA; OR = 1.494, 95% CI 1.212–1.842, *p* ≤ 0.001), heterozygote (CT vs. AA; OR = 1.303, 95% CI 1.119–1.516, *p* = 0.001), dominant (CC + CT vs. AA; OR = 1.341, 95% CI 1.161–1.548, *p* ≤ 0.001), and recessive (CC vs. CT + AA; OR = 1.340, 95% CI 1.119–1.604, *p* = 0.001). Subgroup analyses indicated significant associations in Asian populations, particularly in studies conducted in China and India, while no significant correlations were found in Caucasian populations, including those from Austria. Moreover, the MTHFR A1298C polymorphism did not demonstrate a significant relationship with PTB risk across the studied ethnicities.

**Conclusions:**

The findings indicate a significant association between the MTHFR C677T polymorphism and PTB risk, particularly in Asian and Indian populations, while no significant associations were identified in Caucasian groups. Conversely, the MTHFR A1298C polymorphism appeared to have a negligible impact on PTB risk, underscoring the importance of considering population-specific factors in understanding the genetic epidemiology of PTB.

## Introduction

Preterm birth (PTB) is a multifaceted syndrome traditionally defined by gestational age at birth (under 37 weeks), a classification that presents significant limitations in clinical application and may account for the slow progress in identifying effective, cause-specific interventions [1]. Affecting roughly 10% of newborns worldwide, PTB is a major contributor to infant mortality and long-term health complications, while also placing emotional and financial burdens on families [2–4]. It is important to recognize the distinctions within PTB, which is categorized into three gestational subgroups: extremely preterm (less than 28 weeks), very preterm (28 to less than 32 weeks), and moderate to late preterm (32 to less than 37 weeks) [5]. Moreover, PTB is divided into iatrogenic cases, which make up about 30–40% and result from medical interventions such as labor induction or cesarean delivery, and spontaneous cases, which occur naturally usually due to factors like preterm labor or ruptured membranes [6, 7]. A functional taxonomy of PTB, based on key conceptual principles, known etiological factors, specific maternal and neonatal clinical phenotypes, and follow-up of growth and development up to two years, can improve understanding and management of the condition [1]. Contributing factors for spontaneous PTB include infections, cervical incompetence, and psychosocial stress, with triggering conditions such as intrauterine infections or multiple pregnancies [8, 9]. In contrast, iatrogenic PTB is typically a result of medical decisions aimed at protecting maternal or fetal health. Given that the clinical characteristics and neonatal outcomes of PTB vary significantly with gestational age, a comprehensive understanding of these distinctions is essential for healthcare providers to manage and prevent PTB effectively [10, 11].

Each year, approximately 15 million infants are born preterm, with 13.4 million in 2020, accounting for 9.9% of live births—a slight decline from 2010 [12]. PTB rates vary significantly by region, with about 1 in 10 births in the United States [13], compared to 10–25% in developing countries [14], especially in Southern Asia and sub-Saharan Africa, where healthcare access and data quality disparities are notable [2, 15]. Multiple factors influence the incidence of PTB, including medical, lifestyle, pregnancy-related, and demographic aspects. Women with a history of PTB face a significantly higher risk of subsequent preterm deliveries. Risk factors include a short cervix, early dilation, and multiple pregnancies, particularly with twins [16]. Maternal age is also a factor, as both very young mothers (under 18) and older mothers (over 35) are at increased risk due to possible health issues like hypertension and diabetes [17]. Other medical conditions, such as infections, diabetes, hypertension, and preeclampsia, heighten the likelihood of PTB [18]. Lifestyle influences, including poor nutrition, substance use (smoking, alcohol, and drugs), high stress, low socioeconomic status, and domestic violence, are additional contributors [19]. Pregnancy-related factors, including a short interpregnancy interval (under six months), vaginal bleeding, and infections like bacterial vaginosis or UTIs, can increase the risk of PTB [20, 21].

The methylenetetrahydrofolate reductase (MTHFR) gene is essential for folate metabolism, which is critical for DNA synthesis, repair, and neurotransmitter production [22]. This gene’s role becomes particularly significant during pregnancy, as adequate folate levels are vital for fetal development and maternal health, especially concerning hyperhomocysteinemia [23]. Variants in the MTHFR gene can impair enzymatic function, leading to altered folate levels and potential adverse outcomes, such as PTB, preeclampsia, and recurrent pregnancy loss [24]. Common polymorphisms, notably C677T and A1298C, have been extensively studied, with the C677T variant being particularly impactful on enzymatic activity. Individuals with the TT genotype typically exhibit reduced enzyme activity, resulting in elevated homocysteine and decreased folate levels [25]. However, research on the association between MTHFR polymorphisms and PTB has produced mixed results. While some studies suggest a significant correlation between the MTHFR C677T variant and increased PTB risk [26], others report inconsistent outcomes, often due to small sample sizes and diverse populations [27]. These inconsistencies can arise from variations in study design, population genetics, environmental factors, and gene-environment interactions. Methodological differences, such as demographic variations and sample sizes, can produce divergent findings, especially among ethnic groups; for instance, the A1298C polymorphism is significantly linked to PTB risk in Asian populations but not elsewhere [28, 29]. The prevalence of MTHFR variants varies between populations, affecting risk assessments. Furthermore, environmental factors like nutrition and lifestyle may interact with genetic predispositions, complicating outcomes [27]. Many studies have small sample sizes, limiting statistical power and highlighting the need for larger, well-structured studies to clarify these associations and account for confounding variables [30]. Additionally, some research may inadequately evaluate the interactions between genetic and environmental factors, potentially underestimating genetic effects on PTB risk [31].

Between 2016 and 2018, three meta-analyses [32–34] examined the relationship between MTHFR variants and PTB, yet significant gaps remain in the literature regarding the overall impact of these genetic factors on PTB risk. This lack of clarity may stem from the heterogeneity of the included studies, which differed in sample size, population characteristics, and methodologies, leading to conflicting results that obscure meaningful associations. Moreover, many studies failed to stratify results by ethnicity or geographical location, despite known differences in allele frequencies and prevalence of MTHFR variants across diverse populations. This meta-analysis aimed to quantitatively evaluate the existing research to clarify these associations and understand the underlying mechanisms, seeking to address previous discrepancies. It hypothesized that MTHFR polymorphisms significantly contribute to increased PTB risk, emphasizing the importance of this research for both filling knowledge gaps and potential clinical implications. By assessing the strength and consistency of the relationship across various populations and identifying moderating factors, the study’s findings can help guide healthcare strategies and improve identification of at-risk populations, ultimately contributing to better outcomes in maternal-fetal medicine for both mothers and infants.

## Materials and methods

### Search strategy

Ethical endorsement was not required for this study, as it involved a bibliographic review and meta-analysis conducted following the Preferred Reporting Items for Systematic Reviews and Meta-Analyses (PRISMA) guidelines. An extensive search was performed across several key online bibliographic databases to identify all available studies examining the correlation between MTHFR polymorphisms and the predisposition to PTB, published from the inception of these databases up to January 1, 2025. The databases prioritized for this search included MEDLINE, PubMed, Cochrane Library, EMBASE, Scopus, Web of Science, and Google Scholar, along with additional resources such as PMC (PubMed Central), Elsevier, Europe PMC, ResearchGate, SciELO, the Chinese National Knowledge Infrastructure (CNKI), Wanfang Data Company, Chaoxing, China/Asia On Demand (CAOD), the Chinese Medical Citation Index (CMCI), Semantic Scholar, the Chinese Biomedical Database (CBD), VIP Information Consultancy Company (VIP), MedRxiv, the Chinese Medical Current Contents (CMCC), Web of Knowledge (WOK), the Scientific Information Database (SID), the Economic and Knowledge Base (EKB), SpringerLink, JSTOR, PsycINFO, ClinicalTrials.gov, and the Weipu Periodical Database. The search strategy utilized a comprehensive approach, combining keywords and MeSH terms such as (“Preterm Birth” OR “Preterm Delivery” OR “Spontaneous Preterm Birth” OR “Preterm Labor” OR “Prematurity”) AND (“Methylenetetrahydrofolate Reductase” OR “MTHFR” OR “Folate Metabolism” OR “Folate Pathway”) AND (“Gene”, “Polymorphism”, “DNA Sequence”, “Single-Nucleotide Polymorphism”, “SNPs”, “Genotype”, “Frequency”, “Mutation”, “Mutant”, “Allele”, “Variation”, “Variant”, “Genetic predisposition”). To ensure a thorough review, we examined the reference lists of all retrieved articles for any relevant studies that might have been overlooked in the initial search. We placed no restrictions on language or publication year, translating non-English articles as necessary for a complete analysis.

### Inclusion and exclusion criteria

Inclusion and exclusion criteria for the study were clearly defined. The inclusion criteria were as follows: (1) Only case-control or cohort studies examining the relationship between MTHFR polymorphisms and the risk of PTB were included; (2) Diagnoses of premature birth occurred between 28 and 37 weeks of gestation; (3) Genotype frequency distributions in both case and control groups were necessary for calculating odds ratios (ORs) with 95% confidence intervals (CIs); (4) Studies provided adequate demographic data on participants; (5) Only studies published up to January 2025 were considered to ensure relevance and timeliness. The exclusion criteria included: (1) Reviews, meta-analyses, abstracts, conference papers, case reports, letters to editors, comments, and duplicates were omitted; (2) Studies lacking a control group or exhibiting inappropriate selection processes were excluded; (3) Articles presenting duplicated content from the same author were omitted; (4) Studies lacking gene frequency data without the possibility of supplementation were excluded; (5) Animal and in vitro studies were not considered. In cases of identical data reported in multiple publications, only the study with the larger sample size or the most recent publication was included in the pooled analysis.

### Data extraction

Two investigators independently reviewed titles, abstracts, and search terms for eligibility based on predetermined inclusion and exclusion criteria. Any disagreements were resolved through discussion or by involving a third researcher, and when necessary, the original authors were contacted via email. During the literature screening, the initial focus was on titles and abstracts to eliminate obviously irrelevant studies, followed by a thorough reading of full texts to determine final inclusion. Key data from eligible literature included the first author’s name, ethnicity (specifically categorized as Asian, Caucasian, African, Hispanic, and Mixed), publication date, genotyping methods, country of origin, total hyperbilirubinemia cases and controls, genotype frequencies for neonatal hyperbilirubinemia cases and healthy controls related to MTHFR polymorphisms, Hardy-Weinberg equilibrium (HWE) test results, and minor allele frequencies (MAFs) in healthy controls. For studies by the same authors, the most recent publication or the one with the largest sample size was chosen for inclusion.

### Quality score assessment

The Newcastle-Ottawa Score (NOS) was utilized to assess study quality in a meta-analysis and evaluate the methodological aspects of observational research. It focused on case selection, group comparability, and exposure determination, each with eight specific criteria. Studies with excellent selection and exposure received one star, while comparability could earn up to two stars. Quality was rated on a nine-star scale, where zero indicated poor quality and nine signified high quality. Studies scoring seven or more were deemed high quality, and those with at least five points were suitable for meta-analysis.

### Statistical analysis

The association between MTHFR polymorphisms and the risk of PTB was assessed through ORs and 95% CIs. A Z-test was employed to analyze the aggregated data, comparing the population mean to the sample mean. The meta-analysis utilized five genetic models: allelic (M vs. W), heterozygote (MW vs. WW), homozygote (MM vs. WW), recessive (MM vs. MW + WW), and dominant (MM + MW vs. WW), where “M” denotes the mutant allele and “W” represents the wild-type allele. To evaluate heterogeneity across studies, several statistical measures were applied, including the Q-value, degrees of freedom (df), I-squared (I²), and Tau-squared (τ²). The Q-value tests the null hypothesis of a common effect size among studies, with a higher value relative to its df indicating greater heterogeneity. The I-squared statistic quantifies the proportion of total variation attributed to heterogeneity rather than random chance, with values over 50% indicating moderate to high heterogeneity, while Tau-squared estimates the variance between studies due to inherent variability. The chi-square test was the primary method for assessing heterogeneity, with a significance level set at *p* < 0.05, and heterogeneity was quantified on a scale of 0 to 100% following Cochrane guidelines. Random-effect models (DerSimonian-Laird method) were applied when I² exceeded 50%, while fixed-effect models (Mantel-Haenszel method) were used otherwise. The Pearson’s chi-square statistic assessed HWE among control subjects, utilizing free online software with a significance threshold of *p* < 0.05 [35]. Sensitivity analysis was conducted by systematically excluding one study at a time to evaluate the robustness of the results. Publication bias was examined using Begg’s test, Egger’s test, and visual inspection of funnel plots for asymmetry, with the trim-and-fill method employed to adjust results if bias was detected. Data synthesis from the primary studies was performed using Comprehensive Meta-Analysis (Version 4.0) software, considering a two-sided p-value of less than 0.05 as statistically significant.

### Trial sequential analysis

Trial Sequential Analysis (TSA) was conducted to examine the association between MTHFR 677 C/T and 1298 A/C polymorphisms and the risk of PTB. Using TSA software, accumulated evidence from the meta-analysis was evaluated, emphasizing the adequacy of sample size and the robustness of the results against potential type I and II errors. A comprehensive literature search across multiple databases ensured the inclusion of relevant studies, from which data were extracted to compute overall ORs with 95% CIs. The required information size (RIS) was calculated based on observed event rates and expected effect sizes, and cumulative z-curves were plotted to determine whether evidence crossed trial sequential monitoring boundaries for benefit or harm, alongside traditional significance thresholds. Heterogeneity among studies was assessed using I² statistics, and random-effects models were applied when significant heterogeneity was present. To address the multiple testing issue common in meta-analyses, a sequential trial design was adopted in the TSA. Ultimately, the results were interpreted in light of the number of trials analyzed, cumulative statistical power, and estimated risks of type I and II errors, providing a comprehensive understanding of the impact of MTHFR polymorphisms on PTB incidence.

## Results

### Characteristics of selected studies

**Fig. 1:**
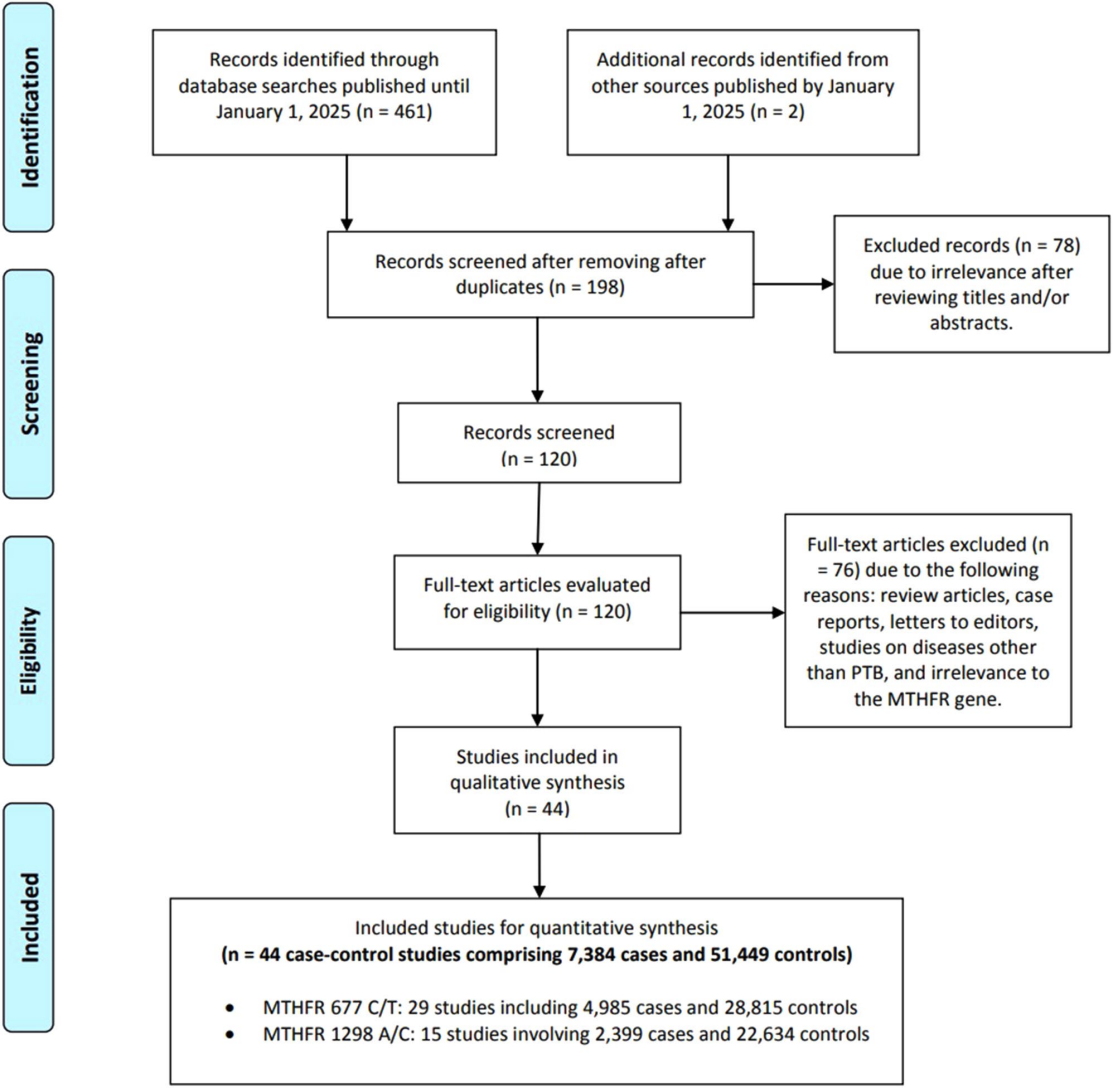
Illustration of the study selection and inclusion process

As illustrated in Fig. 1, a computerized search yielded 463 articles on MTHFR polymorphisms and PTB. After removing duplicates, 198 records were screened, leading to 78 exclusions based on titles and abstracts. Subsequently, 120 full-text articles were evaluated for eligibility, resulting in 76 exclusions due to review articles, case reports, letters to editors, studies on diseases other than PTB, and irrelevance to the MTHFR gene. Ultimately, 44 case-control studies from 28 publications [24, 26, 27, 29, 31, 36–58] in English and Chinese were included, comprising 7,384 cases and 51,449 controls. Of these studies, 29 focused on the MTHFR C677T polymorphism (4,985 cases and 28,815 controls), while 15 analyzed the MTHFR A1298C variant (2,399 cases and 22,634 controls). Detailed data for both polymorphisms can be found in Table 1. The meta-analysis encompassed 15 countries and their ethnic groups, predominantly featuring Asian populations—particularly from China (10 instances), India (5 instances), and Korea. Moreover, Caucasian representation included studies from Austria (4 instances), Norway (2), the USA (6), Denmark (1), and Turkey (2), while Mexico had a mixed ethnic contribution and African ethnicities were present in 3 USA studies. Various genotyping methods were utilized, including PCR-RFLP, TaqMan, and qRT-PCR, along with techniques like Microarray, SNaPshot, and MassARRAY. The Newcastle-Ottawa Scale (NOS) scores varied, with most studies scoring between 5 and 8. The highest number of studies achieved a score of 7, indicating robust methodologies with adequate selection criteria, comparability of groups, and outcome assessment. Figure 1 illustrates this process.

**Table 1:** Characteristics of studies included in this meta-analysis

First author	Country (Ethnicity)	SOC	Genotyping Technique	Case/Control	Cases					Controls					MAFs	HWE	NOS
					Genotypes			Allele		Genotypes			Allele				
**677 C/T**					**CC**	**CT**	**TT**	**C**	**T**	**CC**	**CT**	**TT**	**C**	**T**			
Resch 2004	Austria(Caucasian)	HB	PCR-RFLP	21/44	12	8	1	32	10	26	16	2	68	20	0.227	0.814	7
Nurk 2004	Norway(Caucasian)	HB	qRT-PCR	774/23,176	405	294	75	1104	444	6522	5496	1158	18,540	7812	0.296	0.997	8
Valdez 2004	Mexico(Mixed)	HB	PCR-RFLP	86/162	14	44	28	72	100	45	86	31	176	148	0.457	0.374	6
Chen 2004	China(Asian)	HB	PCR-RFLP	243/247	48	119	76	215	271	60	97	90	217	277	0.561	0.001	6
Engel 2006	USA(Caucasian)	HB	TaqMan	69/335	30	31	8	91	47	160	138	37	458	212	0.316	0.382	7
Engel 2006	USA(African)	HB	TaqMan	67/238	60	7	0	127	7	197	37	4	431	45	0.095	0.156	7
Stonek 2007	Austria(Caucasian)	HB	Microarray	114/1397	43	52	19	138	90	669	573	155	1911	883	0.316	0.055	8
Uvuz 2009	Turkey(Caucasian)	HB	qRT-PCR	50/50	25	21	4	71	29	32	17	1	81	19	0.190	0.456	6
Gargano 2009	USA(Caucasian)	HB	PCR-RFLP	152/408	62	90	-	-	-	174	234	-	-	-	-	-	7
Gargano 2009	USA(African)	HB	PCR-RFLP	71/328	57	14	-	-	-	265	63	-	-	-	-	-	7
Yang 2010	China(Asian)	HB	PCR-RFLP	101/171	27	42	32	96	106	69	72	30	210	132	0.386	0.144	6
Hiltunen 2011	Finland(Caucasian)	HB	PCR-RFLP	323/748	180	126	17	486	160	423	289	36	1135	361	0.241	0.131	7
Lykke 2012	Denmark(Caucasian)	HB	qRT-PCR	619/1842	311	254	54	876	362	906	793	143	2605	1079	0.293	0.091	8
Du 2013	China(Asian)	HB	TaqMan	220/220	59	91	70	209	231	89	93	38	271	169	0.384	0.114	6
Nan 2015	China(Asian)	HB	PCR-RFLP	108/108	26	44	38	96	120	40	49	19	129	87	0.403	0.554	6
Tiwari 2015	India(Asian)	HB	PCR-RFLP	209/194	148	49	12	345	73	170	20	4	360	28	0.072	0.001	6
Mei 2015	China(Asian)	HB	PCR-RFLP	81/102	22	34	25	78	84	41	43	18	125	79	0.387	0.259	7
Wang 2015	China(Asian)	HB	SNaPshot	315/188	108	142	65	358	272	53	100	35	206	170	0.452	0.312	6
Huang 2016	China(Asian)	HB	PCR-RFLP	200/200	141	50	9	332	68	175	19	6	369	31	0.078	≤ 0.001	7
Hwang 2018	Korea(Asian)	HB	PCR-RFLP	98/128	30	57	11	117	79	46	55	27	147	109	0.426	0.0170	6
Karasoulos 2022	Austria(Caucasian)	HB	MassARRAY	141/2785	64	68	9	196	86	1335	1168	282	3838	1732	0.311	0.260	7
Mengmeng 2022	China(Asian)	HB	PCR	125/96	34	58	33	126	124	41	39	16	121	71	0.370	0.208	6
Wang 2022	China(Asian)	HB	MassARRAY	27/691	3	24	-	-	-	86	605	-	-	-	-	-	5
Panikar 2023	India(Asian)	HB	PCR-RFLP	150/150	108	35	7	251	49	130	17	3	277	23	0.077	0.014	7
Panigrahi 2023	India(Asian)	HB	TaqMan	44/8	27	13	4	67	21	6	1	1	13	3	0.188	0.095	5
Rathod 2023	India(Asian)	HB	PCR-RFLP	100/100	36	61	3	133	67	56	44	0	156	44	0.220	0.004	6
Huang 2023	China(Asian)	HB	PCR	54/676	31	16	7	78	30	404	219	53	1027	325	0.240	0.003	6
Wu 2024	China(Asian)	HB	TaqMan	373/3973	194	151	28	539	207	2094	1584	295	5772	2174	0.274	0.848	8
Pavlic 2024	Slovenia(Caucasian)			50/50	21	23	6	65	35	18	27	5	63	37	0.370	0.262	5
**1298 A/C**					**AA**	**AC**	**CC**	**A**	**C**	**AA**	**AC**	**CC**	**A**	**C**			
Nurk 2004	Norway(Caucasian)	HB	qRT-PCR	774/13,166	354	347	73	1055	493	6003	5764	1399	17,770	8562	0.325	0.780	8
Engel 2006	USA(Caucasian)	HB	TaqMan	69/335	23	30	16	76	62	123	140	72	386	284	0.424	0.008	7
Engel 2006	USA(African)	HB	TaqMan	67/238	2	27	38	31	103	24	95	119	143	333	0.700	0.437	7
Uvuz 2009	Turkey(Caucasian)	HB	qRT-PCR	50/50	17	27	6	61	39	24	21	5	69	31	0.310	0.897	6
Gargano 2009	USA(Caucasian)	HB	PCR-RFLP	152/408	70	82	-	-	-	189	219	-	-	-	-	-	7
Gargano 2009	USA(African)	HB	PCR-RFLP	71/328	48	23	-	-	-	220	108	-	-	-	-	-	7
Du 2013	China(Asian)	HB	TaqMan	220/220	155	65	0	375	65	136	80	4	352	88	0.200	0.043	6
Nan 2015	China(Asian)	HB	PCR-RFLP	108/108	71	36	1	178	38	61	38	9	160	56	0.259	0.383	6
Hwang 2018	Korea(Asian)	HB	PCR-RFLP	98/128	69	29	0	167	29	84	40	4	208	48	0.188	0.771	6
Ling 2018	China(Asian)	HB	PCR-RFLP	154/144	88	60	6	236	72	109	26	9	244	44	0.153	≤ 0.001	7
Wang 2022	China(Asian)	HB	MassARRAY	27/691	22	5	-	-	-	546	145	-	-	-	-	-	5
Karasoulos 2022	Austria(Caucasian)	HB	MassARRAY	142/2787	59	68	15	186	98	1352	1197	238	3901	1673	0.300	0.238	8
Panigrahi 2023	India(Asian)	HB	TaqMan	44/8	15	18	11	48	40	4	1	3	9	7	0.438	0.034	5
Wu 2024	China(Asian)	HB	TaqMan	373/3973	217	138	18	572	174	2349	1414	210	6112	1834	0.231	0.882	7
Pavlic 2024	Slovenia(Caucasian)			50/50	21	23	6	65	35	22	25	3	69	31	0.310	0.232	5

### HWE

The analysis of HWE for the MTHFR C677T and A1298C polymorphisms across various studies revealed a wide range of HWE P-values, indicating significant variability in genotype distributions among different populations. For the C677T polymorphism, the majority of studies reported HWE P-values above the conventional threshold of 0.05, suggesting that the genotype frequencies in these cohorts were in equilibrium, particularly in Caucasian populations such as those from Austria, Norway, and the USA. However, several studies from Asian populations, including those conducted in China, demonstrated notably low HWE P-values, indicating significant deviations from equilibrium in these groups. In contrast, for the A1298C polymorphism, the results were more consistent, with many studies reporting HWE P-values above 0.05, particularly in Caucasian and Asian populations alike, although some studies, such as those from the USA and China, exhibited lower P-values. These findings highlight the necessity for considering ethnic and regional variations when evaluating the genetic epidemiology of MTHFR polymorphisms and their association with PTB risk.

### Quantitative data synthesis

Tables 2 and 3 present risk estimates for the association between MTHFR polymorphisms and PTB across the overall population, various subgroups, and genetic models.

**Table 2:** Results of the association of MTHFR C677T polymorphism with preterm birth in overall population, and by ethnicity and country

Subgroup	Genetic model	Type of model	Heterogeneity				Tau				Odds Ratio				Publication bias	
			Q-Value	df(Q)	*P*_H_	I^2^(%)	Tau	SD	Variance	Tau	OR	95% CI	Z_test_	*P*_OR_	*P*_Beggs_	*P*_Eggers_
Overall	C vs. A	Random	99.599	25	≤ 0.001	74.89	0.064	0.033	0.001	0.253	1.303	1.151–1.475	4.188	≤ 0.001	0.047	0.003
	CC vs. AA	Random	53.033	25	0.001	52.86	0.123	0.077	0.006	0.350	1.494	1.212–1.842	3.761	≤ 0.001	0.427	0.057
	CA vs. AA	Random	71.043	25	≤ 0.001	64.81	0.080	0.046	0.002	0.282	1.303	1.119–1.516	3.413	0.001	0.402	0.001
	CC + CA vs. AA	Random	85.605	28	≤ 0.001	67.29	0.083	0.045	0.002	0.289	1.341	1.161–1.548	3.999	≤ 0.001	0.284	0.001
	CC vs. CA + AA	Random	47.493	25	0.004	47.36	0.082	0.055	0.003	0.287	1.340	1.119–1.604	3.181	0.001	0.659	0.205
Asians	C vs. A	Random	61.793	14	≤ 0.001	77.34	0.102	0.058	0.003	0.319	1.501	1.238–1.820	4.136	≤ 0.001	0.047	0.015
	CC vs. AA	Random	33.301	14	0.003	57.96	0.188	0.134	0.018	0.433	1.744	1.279–2.378	3.517	≤ 0.001	0.766	0.146
	CA vs. AA	Random	40.533	14	≤ 0.001	65.46	0.127	0.084	0.007	0.356	1.555	1.230–1.967	3.686	≤ 0.001	0.552	0.028
	CC + CA vs. AA	Random	46.652	15	≤ 0.001	67.84	0.130	0.081	0.007	0.361	1.632	1.301–2.049	4.228	≤ 0.001	0.558	0.037
	CC vs. CA + AA	Random	35.384	14	0.001	60.43	0.163	0.114	0.013	0.404	1.490	1.118–1.984	2.724	0.006	0.843	0.213
Caucasians	C vs. A	Fixed	9.683	8	0.288	17.37	0.003	0.009	0.00	0.056	1.015	0.944–1.092	0.410	0.682	0.348	0.132
	CC vs. AA	Fixed	7.511	8	0.483	0.00	0.00	0.043	0.002	0.00	1.108	0.934–1.314	1.175	0.240	0.602	0.478
	CA vs. AA	Fixed	11.951	8	0.153	33.05	0.015	0.024	0.001	0.121	0.966	0.873–1.069	-0.672	0.051	0.465	0.527
	CC + CA vs. AA	Fixed	10.216	9	0.333	11.90	0.003	0.014	0.00	0.058	0.993	0.907–1.087	-0.147	0.883	0.283	0.054
	CC vs. CA + AA	Fixed	6.345	8	0.609	0.00	0.00	0.039	0.002	0.00	1.127	0.956–1.328	1.425	0.154	0.754	0.758
China	C vs. A	Random	41.414	9	≤ 0.001	78.27	0.085	0.057	0.003	0.291	1.424	1.154–1.756	3.302	0.001	0.210	0.017
	CC vs. AA	Random	24.345	9	0.004	63.03	0.170	0.134	0.018	0.412	1.764	1.267–2.457	3.362	0.001	0.720	0.076
	CA vs. AA	Random	24.757	8	0.002	67.68	0.115	0.095	0.009	0.339	1.340	1.013–1.773	2.050	0.040	0.465	0.175
	CC + CA vs. AA	Random	31.245	10	0.001	67.99	0.113	0.085	0.007	0.337	1.475	1.144–1.901	2.999	0.003	0.876	0.098
	CC vs. CA + AA	Random	23.992	9	0.004	62.48	0.128	0.102	0.010	0.358	1.536	1.149–2.054	2.898	0.004	0.720	0.076
India	C vs. A	Fixed	2.213	3	0.529	0.00	0.00	0.067	0.004	0.00	2.189	1.675–2.860	5.737	≤ 0.001	1.000	0.590
	CC vs. AA	Fixed	1.790	3	0.617	0.00	0.00	0.634	0.403	0.00	2.991	1.347–6.641	2.691	0.007	1.000	0.987
	CA vs. AA	Fixed	0.441	3	0.932	0.00	0.00	0.106	0.011	0.00	2.479	1.773–3.466	5.309	≤ 0.001	1.000	0.802
	CC + CA vs. AA	Fixed	0.548	3	0.908	0.00	0.00	0.093	0.009	0.00	2.550	1.858–3.499	5.799	≤ 0.001	0.734	0.442
	CC vs. CA + AA	Fixed	1.694	3	0.638	0.00	0.00	0.628	0.394	0.00	2.460	1.112–5.444	2.222	0.026	1.000	0.881
Austria	C vs. A	Fixed	3.732	2	0.155	46.40	0.029	0.065	0.004	0.169	1.153	0.958–1.387	1.505	0.132	1.000	0.981
	CC vs. AA	Fixed	5.170	2	0.075	61.31	0.310	0.567	0.321	0.557	1.259	0.814–1.948	1.034	0.301	1.000	0.833
	CA vs. AA	Fixed	0.387	2	0.824	0.00	0.00	0.066	0.004	0.00	1.279	0.985–1.660	1.849	0.065	1.000	0.795
	CC + CA vs. AA	Fixed	1.490	2	0.475	0.00	0.00	0.060	0.004	0.00	1.256	0.979–1.612	1.790	0.073	1.000	0.952
	CC vs. CA + AA	Fixed	4.916	2	0.086	59.31	0.258	0.485	0.235	0.508	1.121	0.745–1.688	0.549	0.583	1.000	0.824

**Table 3:** Results of the association of MTHFR A1298C polymorphism with preterm birth in overall population, and by ethnicity and country

Subgroup	Genetic model	Type of model	Heterogeneity				Tau				Odds ratio			
			Q-Value	df(Q)	*P*_H_	I^2^(%)	Tau	SD	Variance	Tau	OR	95% CI	Z_test_	*P*_OR_
Overall	C vs. A	Random	23.215	11	0.016	52.61	0.026	0.025	0.001	0.161	1.039	0.902–1.197	0.528	0.597
	CC vs. AA	Fixed	16.448	11	0.125	33.12	0.080	0.115	0.013	0.283	0.975	0.804–1.182	-0.258	0.796
	CA vs. AA	Random	25.108	11	0.009	56.18	0.055	0.052	0.003	0.234	1.146	0.935–1.404	1.309	0.191
	CC + CA vs. AA	Random	25.640	14	0.029	45.39	0.035	0.034	0.001	0.186	1.071	0.913–1.257	0.845	0.398
	CC vs. CA + AA	Fixed	13.628	11	0.254	19.28	0.030	0.068	0.005	0.173	0.946	0.794–1.127	-0.624	0.533
Asians	C vs. A	Random	15.372	5	0.009	67.47	0.079	0.084	0.007	0.281	0.906	0.680–1.216	-0.643	0.520
	CC vs. AA	Fixed	7.699	5	0.174	35.05	0.260	0.501	0.251	0.510	0.766	0.503–1.166	-1.245	0.213
	CA vs. AA	Random	19.653	5	0.001	74.55	0.166	0.165	0.027	0.408	1.125	0.748–1.691	0.566	0.571
	CC + CA vs. AA	Random	18.313	6	0.005	67.23	0.122	0.123	0.015	0.350	0.999	0.709–1.406	-0.008	0.993
	CC vs. CA + AA	Fixed	7.177	5	0.208	30.33	0.202	0.442	0.196	0.450	0.701	0.464–1.060	-1.685	0.092
Caucasians	C vs. A	Fixed	4.621	4	0.328	13.44	0.003	0.018	0.00	0.058	1.028	0.935–1.130	0.565	0.572
	CC vs. AA	Fixed	4.127	4	0.389	3.074	0.003	0.079	0.006	0.059	1.011	0.812–1.258	0.094	0.925
	CA vs. AA	Fixed	3.138	4	0.535	0.00	0.00	0.033	0.001	0.00	1.076	0.942–1.229	1.075	0.283
	CC + CA vs. AA	Fixed	4.177	5	0.524	0.00	0.00	0.023	0.001	0.00	1.055	0.936–1.190	0.880	0.379
	CC vs. CA + AA	Fixed	2.990	4	0.559	0.00	0.00	0.066	0.004	0.00	0.973	0.791–1.196	-0.261	0.794
China	C vs. A	Random	14.453	3	0.002	79.24	0.107	0.119	0.014	0.327	0.930	0.644–1.342	-0.389	0.697
	CC vs. AA	Random	6.270	3	0.099	52.15	0.428	0.742	0.551	0.654	0.783	0.505–1.214	-1.093	0.274
	CA vs. AA	Random	17.605	3	0.001	82.95	0.203	0.219	0.04	0.451	1.130	0.690–1.850	0.484	0.628
	CC + CA vs. AA	Random	16.995	4	0.002	76.46	0.157	0.166	0.027	0.396	1.003	0.662–1.521	0.016	0.988
	CC vs. CA + AA	Random	5.885	3	0.117	49.02	0.370	0.677	0.458	0.608	0.739	0.479–1.138	-1.373	0.170

**MTHFR C677T**: In a study investigating the association of the MTHFR C677T polymorphism with PTB, significant findings were reported for various genetic models across different populations. Pooled results demonstrated a significant association between the MTHFR C677T polymorphism and PTB under five genetic models: allele (C vs. T; OR = 1.303, 95% CI 1.151–1.475, *p* ≤ 0.001), homozygote (CC vs. AA; OR = 1.494, 95% CI 1.212–1.842, *p* ≤ 0.001), heterozygote (CT vs. AA; OR = 1.303, 95% CI 1.119–1.516, *p* = 0.001), dominant (CC + CT vs. AA; OR = 1.341, 95% CI 1.161–1.548, *p* ≤ 0.001), and recessive (CC vs. CT + AA; OR = 1.340, 95% CI 1.119–1.604, *p* = 0.001) Figure e 2 shows Forest plots illustrating the link between the MTHFR C677T polymorphism and PTB risk globally across various genetic models. In Asian populations, the allele model yielded an OR of 1.501 (95% CI 1.238–1.820, *p* ≤ 0.001), with homozygote (CC vs. AA) showing an OR of 1.744 (95% CI 1.279–2.378, *p* ≤ 0.001) and heterozygote (CA vs. AA) an OR of 1.555 (95% CI 1.230–1.967, *p* ≤ 0.001). In contrast, Caucasian populations showed no significant associations, as indicated by an allele model OR of 1.015 (95% CI 0.944–1.092, *p* = 0.682). Notably, significant associations were found in China (allele model OR = 1.424, *p* = 0.001) and India (allele model OR = 2.189, *p* ≤ 0.001), while no significant associations were observed in Austria. These findings suggest a population-dependent association of the MTHFR polymorphism with PTB, particularly pronounced in Asian and Indian cohorts.

**Fig. 2:**
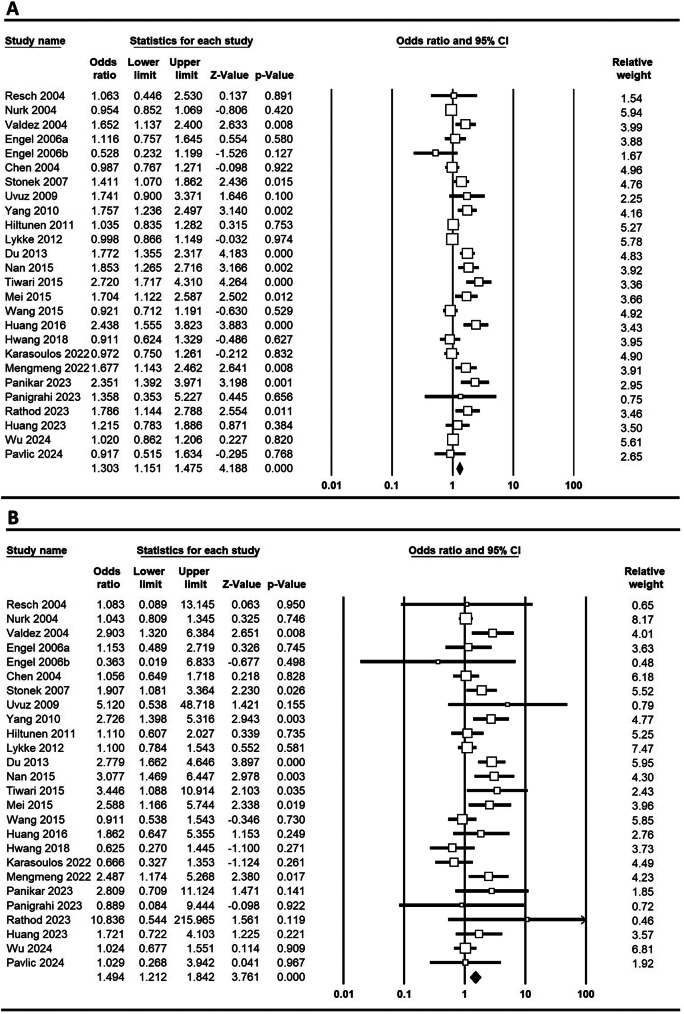

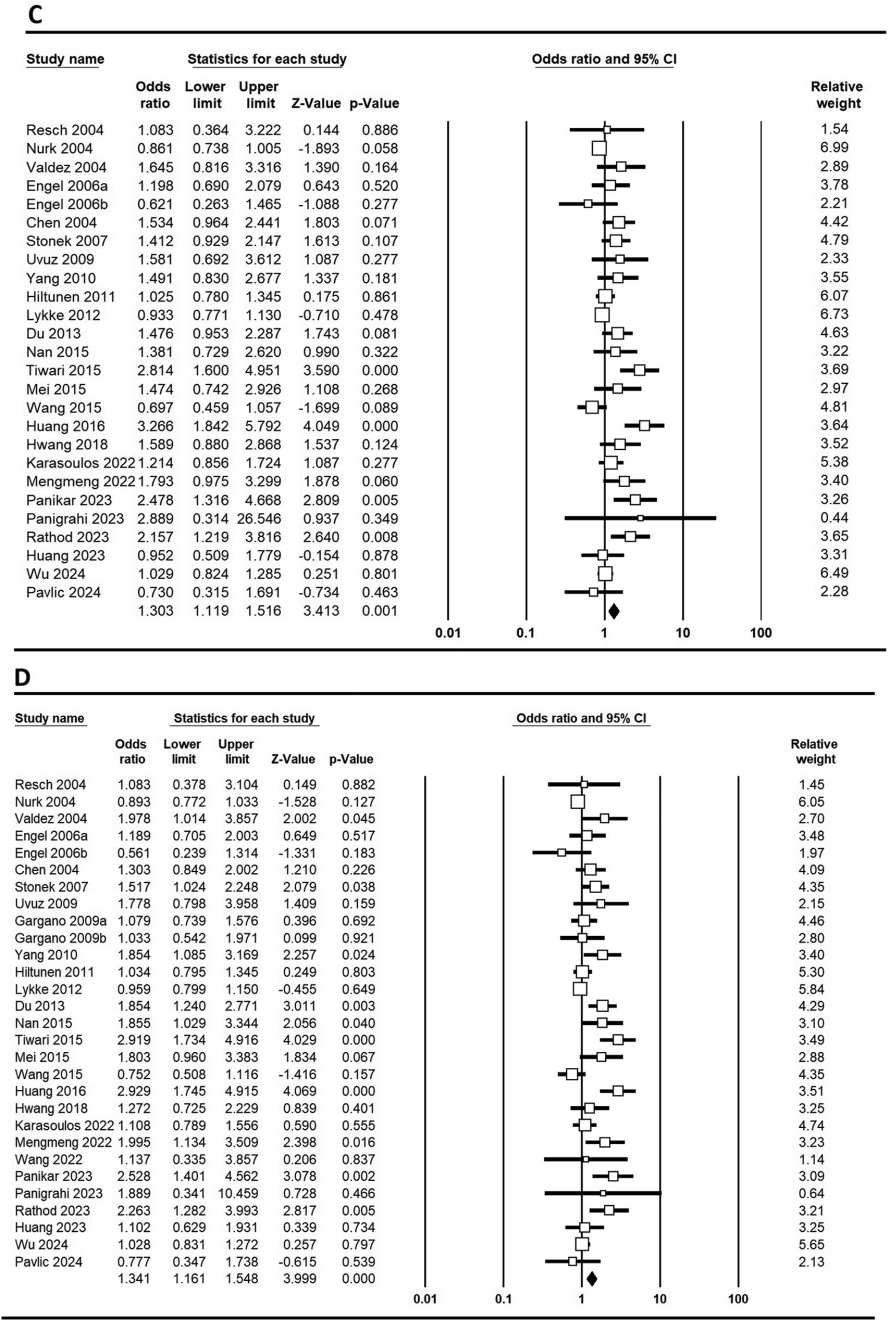

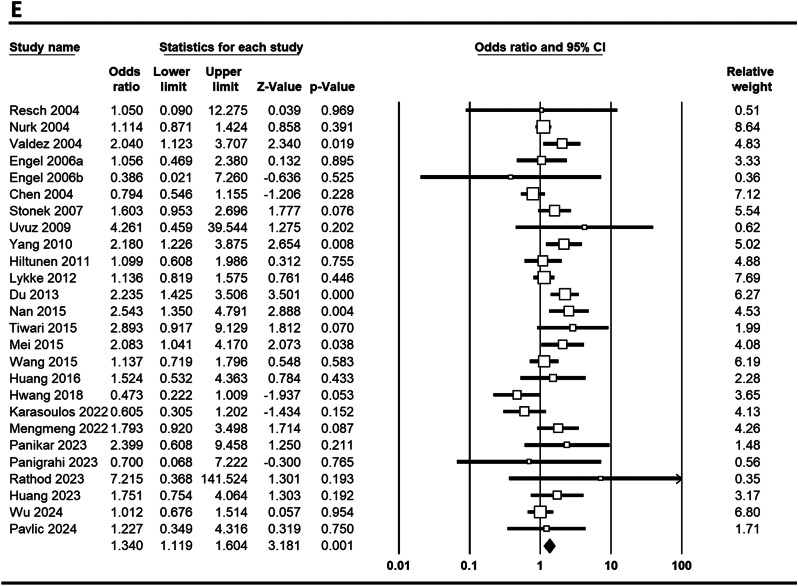
Forest plots depicting the association between the MTHFR C677T polymorphism and the risk of PTB in the global population: **A**: comparison of alleles (C vs. T), **B**: homozygous comparison (CC vs. AA), **C**: heterozygous comparison (CT vs. AA), **D**: dominant model (CC + CT vs. AA), **E**: recessive model (CC vs. CT + AA)

**MTHFR A1298C**: Pooled results indicated no significant association between the MTHFR A1298C polymorphism and PTB across various genetic models: allele (C vs. A; OR = 1.039, 95% CI 0.902–1.197, *p* = 0.597), homozygote (CC vs. AA; OR = 0.975, 95% CI 0.804–1.182, *p* = 0.796), heterozygote (CA vs. AA; OR = 1.146, 95% CI 0.935–1.404, *p* = 0.191), dominant (CC + CA vs. AA; OR values not provided), and recessive (CC vs. CA + AA; OR values not provided). In subgroup analyses, no significant associations were observed among Asians with respective OR of 0.906 (allele model, 95% CI 0.680–1.216, *p* = 0.520), 0.766 (homozygote, 95% CI 0.503–1.166, *p* = 0.213), and 1.125 (heterozygote, 95% CI 0.748–1.691, *p* = 0.571). Caucasians also displayed near neutrality in the allele model (C vs. A; OR = 1.028, 95% CI 0.935–1.130, *p* = 0.572). Notably, the China subgroup revealed an OR of 0.930 (allele model, 95% CI 0.644–1.342, *p* = 0.697) without significant associations in other models. Overall, these findings suggest that the MTHFR A1298C polymorphism is not significantly associated with the risk of PTB across the different ethnic groups and populations studied.

### Heterogeneity test

The analysis of heterogeneity in the association between the MTHFR C677T polymorphism and PTB revealed considerable variability across genetic models and subgroups. In the overall population, a Q-value of 99.599 with a p-value ≤ 0.001 and an I² of 74.89% indicated significant heterogeneity. Subgroup analyses by ethnicity demonstrated further differences, particularly among Asians, who had a Q-value of 61.793 (*p* ≤ 0.001, I² = 77.34%), suggesting substantial variability. In contrast, the Caucasian subgroup exhibited lower heterogeneity, with Q-values between 6.345 and 11.951, all yielding non-significant p-values (≥ 0.153) and I² below 33.05%. The Chinese subgroup also showed significant heterogeneity, with Q-values ranging from 23.992 to 41.414, all significant (*p* ≤ 0.004) and I² values indicating substantial variability (62.48–78.27%). Moreover, the analysis of the MTHFR A1298C polymorphism demonstrated notable heterogeneity across genetic models and subgroups, with the overall population showing moderate heterogeneity in the Random model for C vs. A (Q = 23.215, I²=52.61%, *p* = 0.016). In the CC vs. AA model, a Fixed model indicated low to moderate heterogeneity (Q = 16.448, I²=33.12%, *p* = 0.125), while a higher heterogeneity was observed for CA vs. AA (Q = 25.108, I²=56.18%, *p* = 0.009). Ethnic subgroup analyses reflected varying heterogeneity levels, particularly among Asians for the CA vs. AA comparison (Q = 19.653, I²=74.55%, *p* = 0.001). Conversely, the Caucasian subgroup showed minimal heterogeneity across models, with Q-values from 2.990 to 4.621 and consistently 0% I². The China subgroup also revealed significant heterogeneity for the C vs. A comparison (Q = 14.453, I²=79.24%, *p* = 0.002), highlighting ethnic differences in the association of MTHFR polymorphisms with PTB. These findings emphasize the complexities and varying degrees of heterogeneity in genetic associations related to PTB across different populations and models.

### Publication bias

The assessment of publication bias for the MTHFR C677T and A1298C polymorphisms indicates varying levels of bias across different genetic models and ethnic subgroups. For the MTHFR C677T polymorphism, significant publication bias was identified in the overall population for the C vs. A (PBeggs = 0.047, PEggers = 0.003) and CA vs. AA models (PBeggs = 0.402, PEggers = 0.001). Ethnic analysis showed that Asians exhibited publication bias in both models (C vs. A: PBeggs = 0.047, PEggers = 0.015; CA vs. AA: PBeggs = 0.552, PEggers = 0.028), while Caucasians showed no significant bias. At the country level, China had bias in the C vs. A model (PBeggs = 0.210, PEggers = 0.017), with India and Austria showing no signs of bias. In contrast, the MTHFR A1298C polymorphism showed no significant publication bias in the overall population for the C vs. A model (PBeggs = 0.945, PEggers = 0.593), though notable bias was observed in the CA vs. AA model (PBeggs = 0.086, PEggers = 0.189). Significant bias was found among Asians for the CC vs. AA (PBeggs = 0.259, PEggers = 0.046) and CC vs. CA + AA models (PBeggs = 0.132, PEggers = 0.006). Minimal bias was noted in Caucasians, while China exhibited no significant bias across all models.

### Publication Bias assessment using Duval and Tweedie’s trim and fill method

**Fig. 3:**
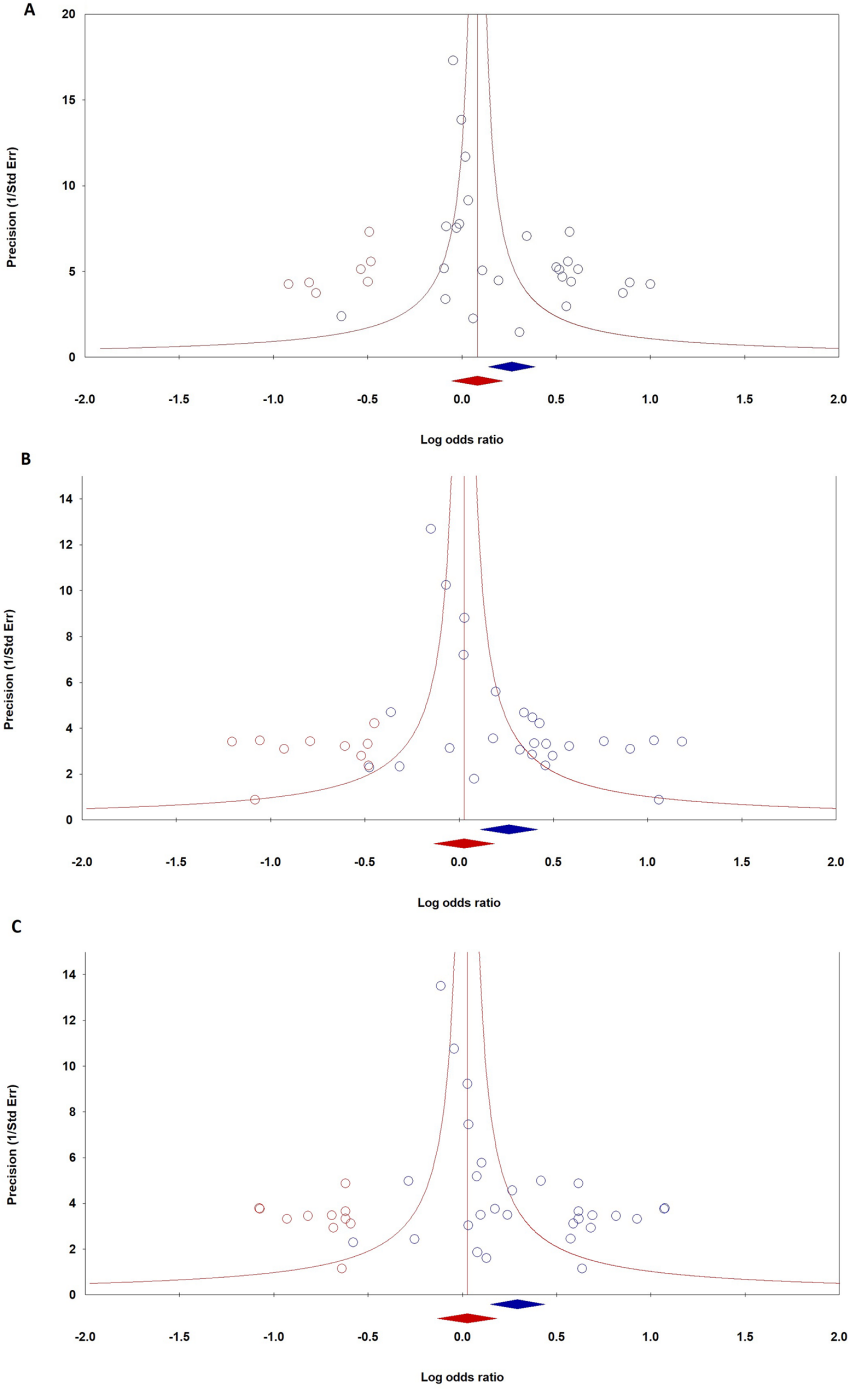
Begg’s funnel plots assessing publication bias regarding the MTHFR C677T polymorphism and PTB risk in the global population: **A**: comparison of alleles (C vs. T), **B**: heterozygous comparison (CT vs. AA), **C**: dominant model (CC + CT vs. AA)

Duval and Tweedie’s trim and fill method assessed publication bias related to the MTHFR C677T polymorphism and PTB, as shown in Fig. 3A-C. The analysis revealed a point estimate of 1.13820 for fixed effects in the allele model (C vs. A), with a CI of 1.07796 to 1.20180, which decreased to 1.09184 after adjustment. For random effects, the observed point estimate was 1.30331, adjusting to 1.17004. In the heterozygote model (CA vs. AA), fixed effects showed a point estimate of 1.10313, adjusted to 1.02659 after trimming seven studies, whereas random effects indicated a point estimate of 1.30266. The dominant model (CC + CA vs. AA) produced an observed fixed effects estimate of 1.13890, adjusting to 1.06448, with random effects showing an estimate of 1.34091, adjusted to 1.15274. The analysis indicated significant heterogeneity across all models with high Q values, emphasizing the need to address variability and potential publication bias in systematic reviews and meta-analyses. Overall, these findings highlight the impact of study trimming on fixed and random effects estimates, with original observed point estimates being higher than adjusted values, suggesting potential publication bias influence on preliminary results.

### Sensitivity analysis

A sensitivity analysis was performed to assess the robustness of the association between the MTHFR C677T and A1298C polymorphisms and the risk of PTB. This analysis evaluated how the OR changed with the exclusion of specific studies or datasets. For the MTHFR C677T polymorphism, the overall random model OR of 1.303 (95% CI: 1.151–1.475) indicated an increased risk of PTB. Notably, substantial heterogeneity was observed (I² = 79.21%), suggesting variability among study results. Excluding studies with high heterogeneity—characterized by wide CIs or smaller sample sizes—led to variations in the OR, which could enhance consistency across findings. In contrast, the MTHFR A1298C polymorphism exhibited a random model OR of 1.039 (95% CI: 0.902–1.197), indicating no significant association with PTB and also showed considerable heterogeneity (I² = 83.18%). Sensitivity analyses that removed studies not adhering to HWE or those suspected of bias were crucial in determining the stability of the association. Significant variations in the OR from these removals, while still retaining statistical significance, pointed to the influential role of certain studies on the overall findings. This underscores the necessity for careful interpretation of the associations of these polymorphisms in the context of predicting PTB risk.

### Trial sequential analysis results

**Fig. 4:**
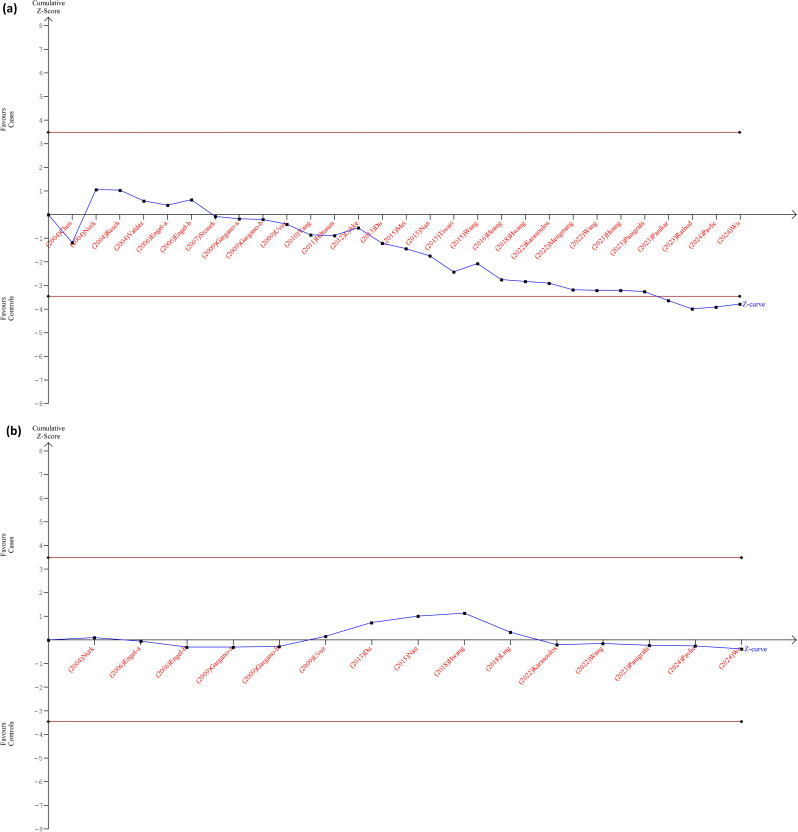
Cumulative Trial Sequential Analysis results for MTHFR polymorphisms in relation to the risk of PTB under a dominant model. **A**: MTHFR C677T; **B**: MTHFR A1298C

The relationship between MTHFR polymorphisms and PTB risk has produced inconsistent findings, necessitating a thorough review of the cumulative data. A TSA was performed to evaluate the reliability and strength of the evidence regarding this association. The pooled effect size for the MTHFR 677 C/T polymorphism suggested a slight increase in PTB risk under a dominant genetic model, with a pooled effect of 1.14 (95% CI: 1.07 to 1.23, Fig. 4A) and a statistically significant p-value of 2.0E-4. However, moderate heterogeneity was noted (I² = 0.67), indicating substantial variability among the studies, supported by a high Q statistic of 85.62 and a Q p-value of 0.0. This suggests that the differences in effect estimates are likely due to inherent study variations rather than random chance. Individual study contributions varied significantly, from 0.14 to 26.2%. Notably, studies by Lykke and Hiltunen showed effect measures near null, while Tiwari reported much higher risk estimates. This discrepancy highlights the need for careful evaluation of study characteristics, methodologies, and populations to clarify the association between MTHFR polymorphisms and PTB risk. Regarding the MTHFR 1298 A/C polymorphism, the TSA indicated a pooled effect estimate of 1.02 (95% CI: 0.93 to 1.12, Fig. 4B) and a p-value of 0.6989, showing no significant association with PTB risk under the dominant genetic model. Considerable heterogeneity was also observed in this analysis, with a Q statistic of 32.96 and a Q p-value of 0.0029, leading to an inconsistency index (I²) of 58%. This complexity underlines the varied findings, with some studies indicating increased risk and others showing a protective effect. Overall, the TSA results highlight the necessity for further research to clarify the conflicting evidence regarding the role of MTHFR polymorphisms in PTB. Strengthening the evidence base will aid in developing future clinical and public health strategies related to PTB risk factors, enhancing our understanding of the genetic influences on PTB outcomes.

### MAF

The analysis of minor allele frequencies (MAFs) for the MTHFR C677T polymorphism revealed notable variations across different ethnic groups. In Caucasian populations, MAFs ranged from 0.190 in Turkey to 0.457 in Mexico, with several studies from Austria, Norway, and the USA showing frequencies around 0.227 to 0.316. In Asian populations, notably higher frequencies were observed, with China exhibiting MAFs between 0.561 and 0.452 across various studies, while Korea presented a frequency of 0.426. Indian samples showed lower frequencies, with values between 0.072 and 0.220. For the MTHFR A1298C polymorphism, Caucasian populations again displayed varying frequencies, with values from 0.300 in Austria to 0.424 in the USA, alongside a significant frequency of 0.700 among African-Americans. Asian populations showed lower MAFs compared to the C677T polymorphism, with frequencies ranging from 0.153 to 0.259 in China, while Indian samples reported a notable frequency of 0.438. Overall, the data suggest substantial ethnic variability in MAFs for both polymorphisms, highlighting the importance of considering genetic diversity in studies related to PTB risk.

## Discussion

The MTHFR C677T polymorphism has attracted considerable research interest due to its effects on folate metabolism and homocysteine levels, both crucial for reproductive health and pregnancy outcomes [59]. The homozygous TT genotype is linked to several adverse outcomes, including lower full-term birth rates and higher risks of gestational diabetes, hypertensive disorders, spontaneous abortions, and fetal abnormalities. Despite the potential complications arising from MTHFR polymorphisms, appropriate medical interventions can enhance live birth rates post-treatment [60]. Notably, paternal MTHFR C677T polymorphisms do not significantly affect embryo quality or neonatal outcomes, highlighting the importance of maternal genetics. The C677T polymorphism is associated with increased homocysteine levels, which may lead to issues such as venous thrombosis and recurrent pregnancy loss. However, the relationship between this polymorphism and pregnancy outcomes is complex; some studies have shown no significant correlation with preterm delivery or other complications after adjusting for factors like age and body mass index, indicating that other variables may also be influential [61–63]. Our analysis of 29 studies involving 4,985 cases and 28,815 controls revealed a significant association between the MTHFR C677T polymorphism and PTB, particularly in Asian and Indian populations, with no significant findings in Caucasian groups. Previous meta-analyses by Chen et al. (2016) [32], Wu et al. (2017) [33], and Fang et al. (2018) [34] examined the association between the MTHFR C677T polymorphism and adverse pregnancy outcomes, each employing different methodologies regarding inclusion criteria and statistical models. Chen et al. investigated both PTB and placental abruption using a random-effects model for ORs with 95% CIs, while Wu et al. focused on PTB and low birth weight, analyzing case-control studies with genotype frequencies and applying a random-effects model with ethnic stratification. Fang et al. specifically analyzed maternal MTHFR polymorphisms related to PTB, calculating pooled ORs across various genetic models within a random-effects framework. These methodological differences may explain the inconsistent findings regarding the impact of the MTHFR C677T polymorphism on pregnancy outcomes. Fang et al. reported a strong correlation between the MTHFR C677T polymorphism and increased PTB risk, particularly in Asian populations [34], while Wu et al. supported this with data from 25 studies indicating significant risk. They stressed the potential benefits of screening for MTHFR mutations in pregnant women, especially in developing countries, as maternal genetics seemed to have a greater influence than neonatal polymorphisms, which showed no significant associations [33]. Chen et al. [32] systematically reviewed 22 studies and found no significant association between the MTHFR C677T polymorphism and PTB or placental abruption. This result remained consistent across different genetic models and highlights the need for further research that includes diverse populations and accounts for potential confounding factors. It underscores the importance of tailored screening and prevention strategies in maternal health, especially in regions where the MTHFR C677T polymorphism may significantly affect PTB.

The MTHFR A1298C variant has been studied for its potential effects on pregnancy outcomes due to its role in folate metabolism, essential for DNA synthesis and repair during pregnancy [53]. Several studies indicate significant improvements in live birth rates among women with the MTHFR C677T and A1298C polymorphisms post-treatment, increasing from 9.4% pre-treatment to 68.7% post-treatment, while abortion rates decreased from 81.2 to 32.1% [61–63]. Additionally, younger maternal age was linked to a 3.76-fold increase in live births among those with the A1298C heterozygous genotype, suggesting better outcomes for younger women [61]. However, pregnancy complications such as preeclampsia and fetal growth restriction were noted in about 18.3% of patients, and the A1298C polymorphism is associated with an increased risk of recurrent pregnancy loss and gestational hypertension [61, 64]. Literature shows mixed results on the link between this polymorphism and adverse pregnancy outcomes, suggesting that environmental factors like folate intake play a significant role. Dietary interventions, particularly a methionine-poor diet al.ongside medical treatment, may enhance pregnancy outcomes for women with MTHFR polymorphisms, highlighting the importance of a comprehensive approach to managing these genetic variations [29, 65]. Our pooled analysis of 15 studies with 2,399 cases and 22,634 controls found no significant link between the MTHFR A1298C variant and PTB risk, indicating minimal genetic impact. A 2018 meta-analysis by Javaheri et al. also found no significant link between the MTHFR A1298C variant and PTB risk across various genetic models [28]. Some studies suggest a potential link between elevated homocysteine levels associated with the MTHFR A1298C variant and PTB [47]; however, larger population-based studies, including one with Croatian and Slovenian women, have not consistently validated this association [27]. Ethnic variations in the data indicate that while overall findings show no significant risk, stratified analyses reveal a significant association in Asian populations, where the allele C appears to offer a protective effect against PTB [31], unlike in Caucasian populations. Some studies indicate that the CC genotype of the A1298C polymorphism may offer protection against preterm delivery, as evidenced by its lower frequency in such cases [31]. Although the A1298C variant does not significantly impair enzymatic activity, it could influence folate metabolism—critical during pregnancy—leading to potential adverse effects if folate intake is low [64]. Clinically, these findings suggest that routine genetic testing for MTHFR A1298C in pregnant women may not effectively predict PTB risk. Researchers generally agree that while ensuring adequate folate levels is essential for pregnancy health, the presence of MTHFR polymorphisms alone does not warrant changes in clinical management or interventions aimed at preventing PTB.

MTHFR polymorphisms, particularly C677T, are significantly linked to PTB in Asian populations, whereas the A1298C variant does not show the same effect. This difference can be attributed to factors like genetic prevalence, metabolic implications, and study methodologies [33, 34]. The C677T variant is more prevalent in Asians, and research demonstrates that individuals with the TT or CT genotypes face a higher risk of PTB compared to those with the CC genotype. Research confirms that the maternal MTHFR C677T polymorphism is associated with a higher risk of PTB across various genetic models [32]. The mechanism is believed to involve the C677T variant, which reduces MTHFR enzyme stability and activity, thereby raising homocysteine levels in the body. High homocysteine concentrations are associated with vascular complications during pregnancy, potentially impairing placental function and increasing PTB likelihood. In contrast, the A1298C polymorphism is less prevalent in Asian populations, with allele frequencies between 1% and 4% [60], which likely diminishes its impact on pregnancy outcomes. Investigations into the A1298C variant have generally shown weak correlations with PTB or other adverse pregnancy outcomes, indicating that its effects are overshadowed by those of C677T [33]. Additionally, geographical distribution plays a significant role, as the C677T variant exhibits considerable variability across regions like China, correlating with differences in pregnancy complications, while the A1298C polymorphism remains relatively stable across populations [60]. These findings have important public health and genetic counseling implications, as understanding the MTHFR C677T polymorphism’s association with PTB can enhance risk assessment and management strategies for pregnant women in Asian populations, whereas the A1298C variant appears less relevant in this context.

The analysis of heterogeneity regarding the MTHFR C677T and A1298C polymorphisms in relation to PTB underscores the complexity of genetic associations across diverse populations. Notably, the pronounced heterogeneity observed within the Asian subgroup, particularly for the MTHFR C677T polymorphism, indicates significant variability in the genetic risk factors influencing PTB, as evidenced by high Q-values and I² percentages. This contrasts sharply with the Caucasian subgroup, where the consistent low heterogeneity suggests a more uniform genetic influence or possibly a lack of associated risk variations, pointing to the intricate interplay between genetic background and environmental factors in different ethnic groups. Moreover, the significant heterogeneity within the Chinese subgroup for both MTHFR polymorphisms further highlights the need for more granular research that takes into account ethnic variations. Such findings not only stress the importance of personalized genetic considerations in clinical settings but also raise questions about the underlying biological mechanisms driving these disparities. Overall, this analysis reinforces the necessity for tailored approaches in future studies aimed at understanding PTB etiology in diverse populations.

### Clinical implications

The pooled data on MTHFR polymorphisms and their association with PTB reveals significant clinical implications for managing pregnancy risks. Notably, the MTHFR C677T variant shows strong associations in populations such as Asians and Indians, suggesting that genetic screening for this polymorphism could help identify women at higher risk for PTB. This targeted approach enables the development of personalized prenatal care strategies, allowing healthcare providers to implement preventive measures or closer monitoring for at-risk groups. Conversely, the lack of significant associations for the MTHFR A1298C polymorphism across various populations indicates that routine screening for this variant may not be necessary. Furthermore, the variations in associations among different populations highlight the importance of culturally sensitive healthcare interventions that address genetic diversity. However, the cost-effectiveness of widespread genetic screening, particularly in developing countries, presents a significant challenge. Although such screening could facilitate targeted interventions, the financial implications must be carefully considered, especially in regions with limited healthcare resources. The economic burden of genetic testing may exceed potential savings from preventing PTB-related complications, which can incur substantial medical costs and long-term care needs for affected infants. For genetic screening to be warranted in developing countries, it must be paired with effective preventive strategies that provide a clear return on investment. Moreover, the necessary infrastructure for implementing genetic testing, including trained healthcare professionals and accessible counseling, may be insufficient in many areas. This situation raises important questions about the practicality of such initiatives, necessitating significant investment in public health initiatives to ensure equitable access. Ultimately, while the potential benefits of personalized prenatal care through genetic screening are considerable, a thorough evaluation of cost-effectiveness and feasibility is essential before widespread clinical adoption can be considered viable, encouraging a more nuanced understanding of genetic factors in PTB and paving the way for improved healthcare outcomes.

### Limitations

To the best of our knowledge, this meta-analysis is the most comprehensive examination of the associations between MTHFR polymorphisms and PTB since the last meta-analysis on this topic published in 2018. However, it does have several notable limitations: (1) The heterogeneity of study populations, influenced by genetic, environmental, and lifestyle variations across different ethnic groups, complicates result interpretation and may confound associations. (2) The observational nature of the included studies restricts causation inference. (3) Reliance on diverse genotyping methodologies raises concerns about the comparability of findings. (4) Inconsistencies in study design, such as varying definitions of PTB and differing control group selections, contribute to potential inconsistencies in outcome assessments. (5) Variability in sample sizes and demographic representation can affect the statistical power and reliability of effect estimates. (6) Confounding factors like maternal age, socio-economic status, and folic acid intake may undermine the validity of the findings. (7) Publication bias tends to favor studies reporting significant results, skewing overall conclusions. (8) Geographical disparities in healthcare access and nutritional status, along with temporal variations in medical practices and diagnostic criteria, add complexity to data synthesis. (9) Potential genetic linkage disequilibrium among MTHFR polymorphisms necessitates careful accounting in analyses to avoid confounding associations with PTB. Collectively, these limitations emphasize the need for standardized methodologies and further research to clarify the associations between MTHFR polymorphisms and PTB.

## Conclusions

Our pooled data show a significant association between the MTHFR C677T polymorphism and PTB, especially in Asian and Indian populations, while no such association was found in Caucasian cohorts. Conversely, the MTHFR A1298C polymorphism did not correlate with PTB risk, indicating its genetic influence is likely minimal. These findings highlight the importance of considering population-specific factors in the genetic epidemiology of PTB, as genetic influences can vary significantly between populations. This underscores the necessity of incorporating genetic backgrounds in research and clinical efforts to understand and manage PTB risks.

## Data Availability

No datasets were generated or analysed during the current study.
